# Influence of apelin-12 on troponin levels and the rate of MACE in STEMI patients

**DOI:** 10.1186/s12872-017-0633-z

**Published:** 2017-07-20

**Authors:** Xhevdet Krasniqi, Blerim Berisha, Masar Gashi, Dardan Koçinaj, Fisnik Jashari, Josip Vincelj

**Affiliations:** 1 Clinical Hospital Dubrava, Zagreb, Republic of Croatia; 2 University Clinical Center of Kosova, Mother Theresa n.n, 10000 Prishtina, Republic of Kosovo

**Keywords:** Apelin, Myocardial infarction, Major adverse cardiac events

## Abstract

**Background:**

During acute myocardial infarction, phosphorylated TnI levels, Ca^2+^ sensitivity and ATPase activity are decreased in the myocardium, and the subsequent elevation in Ca^2+^ levels activates protease I (caplain I), leading to the proteolytic degradation of troponins. Concurrently, the levels of apelin and APJ expression are increased by limiting myocardial injury.

**Methods:**

In this prospective observational study, 100 consecutive patients with ST-elevation acute myocardial infarction were included. Patients meeting the following criteria were included in our study: (1) continuous chest pain lasting for >30 min, (2) observation of ST-segment elevation of more than 2 mm in two adjacent leads by electrocardiography (ECG), (3) increased cardiac troponin I levels, and (4) patients who underwent reperfusion therapy. We evaluated the levels of apelin-12 and troponin I on the first and seventh days after reperfusion therapy in all patients.

**Results:**

Apelin-12 was inversely correlated with troponin I levels (Spearman’s correlation = −0.40) with a *p* value <0.001. There was variability in the apelin values on the seventh day (Kruskal-Wallis test) based on major adverse cardiac events (MACE) (*p* = 0.012). Using ROC curve analyses, a cut-off value of >2.2 for the association of apelin with MACE was determined, and the AUC was 0.71 (95% CI, 0.58–0.84). Survival analysis using the Kaplan-Meier method showed a lower rate of MACE among patients with apelin levels >2.2 (*p* = 0.002), and the ROC curve analysis showed a statistically significant difference in the area under the curve (*p* = 0.004).

**Conclusion:**

The influence of apelin levels on troponin levels in the acute phase of STEMI is inversely correlated, whereas in the non-acute phase, low apelin values were associated with a high rate of MACE.

## Background

After acute myocardial infarction, the left ventricle undergoes a series of changes in shape, size, and thickness, which is referred to as ventricular remodelling; it precedes the development of clinically evident MACE by months to years. The apelin-APJ axis may be up-regulated with good left ventricular remodelling or down-regulated with cardiac troponin degradation and the release of cardiac natriuretic hormones that inhibit the pathophysiological mechanisms responsible for ventricular remodelling [1–3]. The role of hypoxia in the release of apelin, ischaemia reperfusion injury, and the action of apelin in cardiac contractility remains unclear.

### Apelin and the cardiovascular system

The gene for the APJ receptor is located on chromosome 11 and encodes a G-protein coupled receptor that is recognized only by apelin [4, 5]. APJ receptor expression is particularly high in the heart, lung, kidney, cerebellum and vascular endothelium. In the heart, this receptor is expressed on a number of cell types, including the endothelium, smooth muscle and myocytes. The apelin gene is located on the human X chromosome, which, in response to hypoxia, encodes a 77-amino-acid preproprotein that is cleaved by endopeptidases into a biologically active peptide such as apelin-12 [6]. The positive effects of apelin on the cardiovascular system include the regulation of vascular tone, cardiac contractile function and fluid balance [7–10]. Apelin plays a role in diuresis, pituitary hormone release, cardiomyocyte apoptosis and inflammation [11–14].

In acute myocardial infarction, the levels of troponins are predictors of MACE [15–17]. The use of a sensitive troponin I assay improves early diagnosis [18], and these patients are more likely to undergo angiography [19, 20].

### Relationship between apelin and troponin

After the binding of apelin with its receptor, phospholipase C (PLC) is activated and generates inositol trisphosphate (IP3) and diacylglycerol (DAG) from phosphatidyl inositol bisphosphate (PIP2). Diacylglycerol activates protein kinase C (PKC), increasing the activity of the sarcolemmal Na^+^/H^+^ exchanger (NHE). This leads to an elevation in pH, which indirectly increases the intracellular Ca^2+^ concentration through the reverse Na^+^/Ca^2+^ exchanger (NCX). On the other hand, apelin increases intracellular Ca^2+^ concentration via the calcium release channels associated with ryanodine receptors (RyRs) and via the activation of protein kinase C, which decreases the phosphorylation of phospholamban (PLB), reducing the function of the SR Ca^2+^ ATPase (SERCA) [21, 22].

**Fig. 1:**
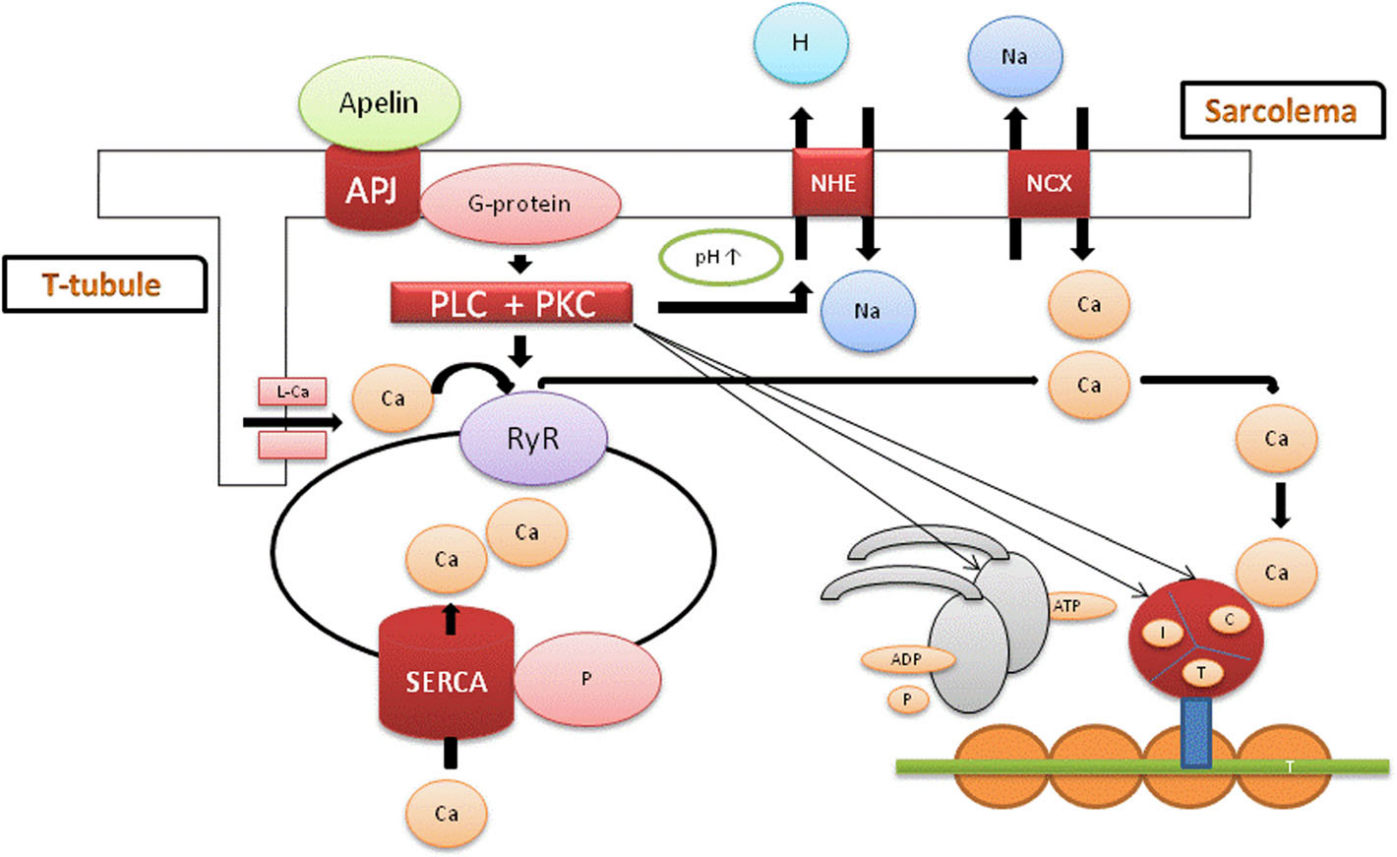
Influence of apelin on troponin

Through protein kinase C, apelin activates its sites on troponin I, thereby regulating Ca^2+^ sensitivity and ATPase activity in the myocardium (Fig. 1) [23, 24].

During ischaemia/infarction, phosphorylated TnI levels, Ca^2+^ sensitivity and ATPase activity are decreased in the myocardium, and subsequently, the elevated Ca^2+^ levels activate protease I (caplain I), which may lead to the proteolytic degradation of troponins [25–28]. Concurrently, the levels of apelin and APJ expression are increased by limiting myocardial injury [1].

The aims of this study were to evaluate the influence of apelin on troponin levels and the rate of major adverse cardiac events (MACE) in STEMI patients.

## Methods

### Study population

In this dual-centre, prospective observational study, one hundred consecutive patients with ST-elevation acute myocardial infarction who presented or were referred to the coronary care unit of our institutions were included. Patients meeting the following criteria were included in our study: (1) continuous chest pain lasting >30 min, (2) the observation of ST-segment elevation of more than 2 mm in two adjacent leads by electrocardiography (ECG), (3) increased cardiac troponin I levels, and (4) patients who underwent reperfusion therapy [29]. We evaluated the levels of apelin-12 and troponin I on the first and seventh days after reperfusion therapy in all patients. The study was approved by the institutional ethics committee of Dubrava University Hospital-Zagreb and the University Clinical Center of Kosova-Pristina. Written informed consent was obtained from all patients before inclusion in the study.

### Patient characterization

Demographic data and clinical history were obtained during hospitalization using a standardized questionnaire. Based on coronarography, the number of diseased vessels, culprit lesions and stenoses was determined. The culprit lesion was defined as the lesion with the highest degree of stenosis or with angiographic signs of endoluminal thrombi and/or plaque rupture. Stenoses ≥50% of the lumen of the left main artery (LMA) or ≥70% of the lumen of major epicardial vessels were considered significant. For the assessment of LV ejection fraction, transthoracic echocardiography was performed in all patients during their hospitalization.

### Laboratory data

Blood samples for the measurement of routine laboratory parameters were collected at admission. On the first and seventh day after reperfusion therapy, blood samples were collected into lavender Vacutainer tubes (Catalogue No. VT-6450) that contain EDTA and can collect up to 7 ml of blood/tube. The blood was transferred from the lavender vacutainer tubes to centrifuge tubes containing aprotinin (0.6TIU/ml of blood) and then centrifuged at 1600 x g for 15 min at 4 °C. The serum was aliquoted and stored at −80 degrees Celsius to prevent degradation. Circulating apelin-12 and troponin I concentrations were analysed using a commercially available enzyme-linked immunosorbent assay (ELISA) kit (Phoenix Pharmaceuticals, Inc) according to the manufacturer’s instructions.

### Statistical analysis

The primary endpoint of the study was the influence of apelin-12 on troponin levels and its association with major adverse cardiac events in STEMI patients. Continuous variables are presented as the mean ± standard deviation or as the median (range), whereas categorical variables are presented as percentages. Spearman’s correlation was used to analyse the degree of association between apelin-12 and troponin I, whereas ROC curve analyses were used to determine a cut-off value of apelin-12 for an association with MACE. Using Kaplan-Meier estimates, we evaluated the association between apelin and future MACE after a follow-up period of 12 months. All statistical analyses were performed using SPSS statistics version 21.

## Results

### Patient characteristics

Baseline characteristics of the study population are displayed in Table 1. The mean age was 60.52 ± 11.50 years old (60% male). Coronary risk factors and laboratory values are presented as a mean, median or percentage. In terms of coronary angiographic findings, the culprit artery was the left anterior descending artery (LAD, 48%), right coronary artery (RCA, 40%) and circumflex (12%), whereas, in terms of vessel disease, 40% of patients had one-vessel disease, 31% had two-vessel disease and 29% had three-vessel disease.

**Table 1:** Baseline characteristics of patients

Parameters	
Age (year), mean (±SD)	60.52 ± 11.50
Gender (male), *n* (%)	60 (60)
Medical history	
Hypertension, *n* (%)	59 (59)
Diabetes mellitus, *n* (%)	19 (19)
Dyslipidemia, *n* (%)	32 (32)
Smoking, *n* (%)	32 (32)
Family history of cardiovascular disease, *n* (%)	20 (20)
Killip class >1, *n* (%)	13 (13)
Ejection fraction, mean (±SD)	50.34 ± 10.20
Laboratory values	
Haemolobin (g/dL), mean (±SD)	13.54 ± 1.39
Creatinine (umol/L), median (range)	92.90 (67.21–124.34)
Apelin 12 on the first day (ng/mL), median (range)	2.98 (0.45–15.25)
Apelin 12 on the seventh day (ng/mL), median (range)	2.33 (0.26–10.90)
Troponin I on the first day (ng/mL), mean (±SD)	54.80 ± 60.99
Troponin I on the seventh day (ng/mL), mean (±SD)	12.43 ± 24.48
Coronary angiographic findings	
Culprit lesion, *n* (%)	
RCA	40 (40)
LAD	48 (48)
LCx	12 (12)
Vessel disease, *n* (%)	
1	40 (40)
2	31 (31)
3	29 (29)

Values are *n* (%), mean (±SD) or median (range)

Abbreviations: *RCA* right coronary artery, *LAD* left anterior descending coronary artery, *LCx* left circumflex coronary artery

### Relationship between apelin and troponin

**Fig. 2:**
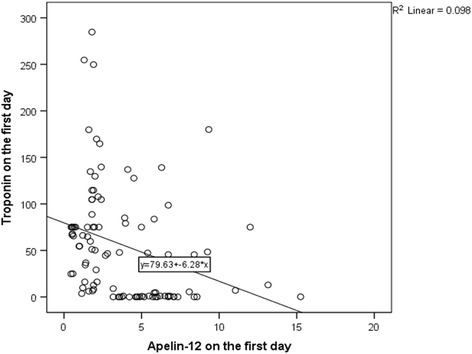
Inverse correlation between apelin-12 and troponin levels in patients with STEMI

The degree of association between apelin-12 and troponin I in the first day of the acute phase of STEMI was analysed with Spearman’s correlation = −0.40 (*p* < 0.001). Based on the regression analysis of the relationship between apelin-12 and troponin I, one variable could be predicted from the other (Fig. 2).

### Apelin and major adverse cardiac events

**Fig. 3:**
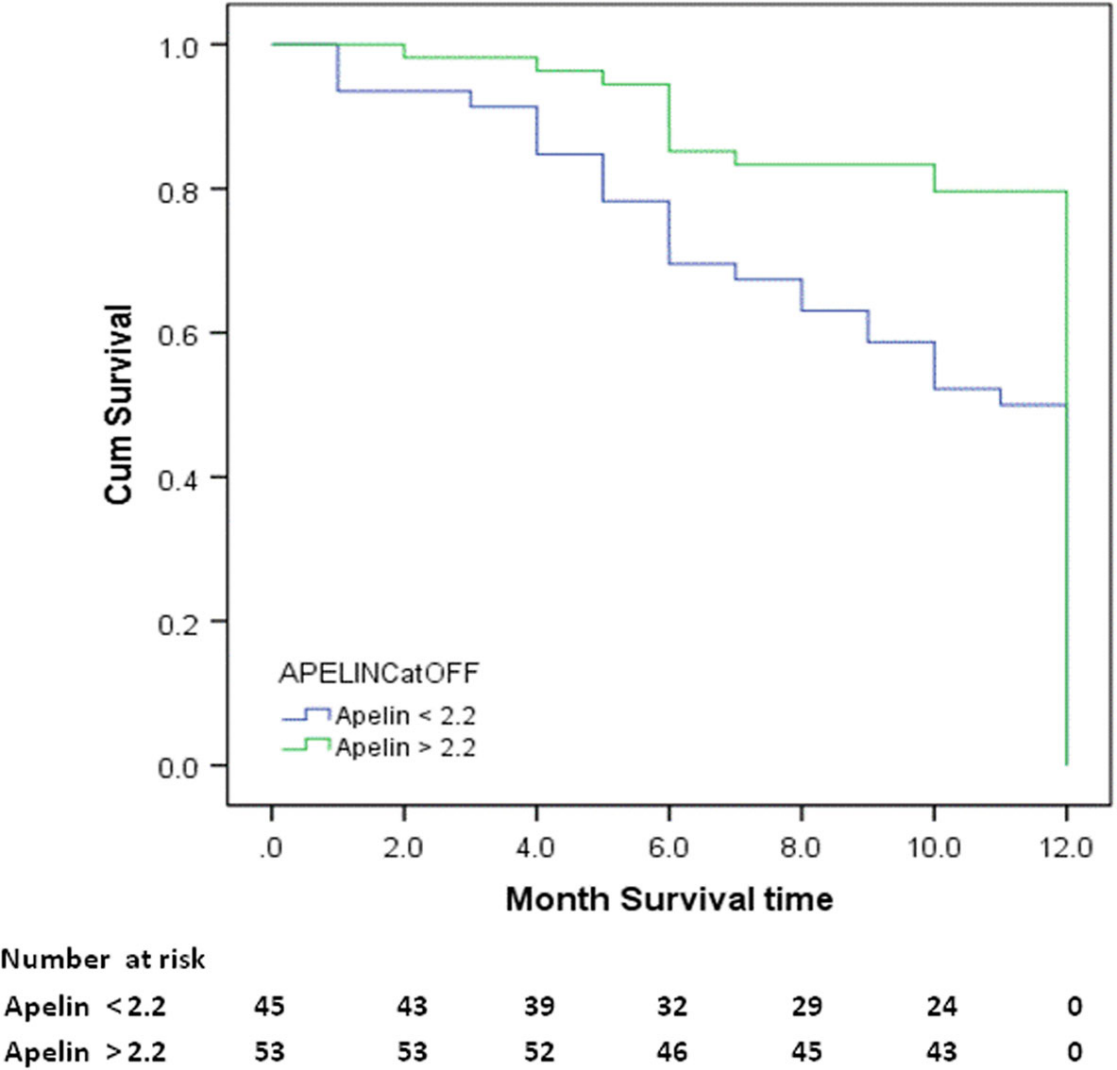
Kaplan-Meier estimates showing lower rates of MACE recurrence among patients with apelin levels >2.2 compared to lower apelin levels <2.2 (*p* = 0.002)

There was variability in the apelin values on the seventh day (Kruskal-Wallis test) in relation to major adverse cardiac events (MACE) that was significantly different (*p* < 0.012). Kaplan-Meier curves were used to show the number of MACE and the proportion of patients that survived at each event time point based on the cut-off value of apelin-12 on the seventh day (2.2 ng/mL) (Fig. 3). The log-rank test for the difference in survival resulted in a *p* value of 0.002.

**Fig. 4:**
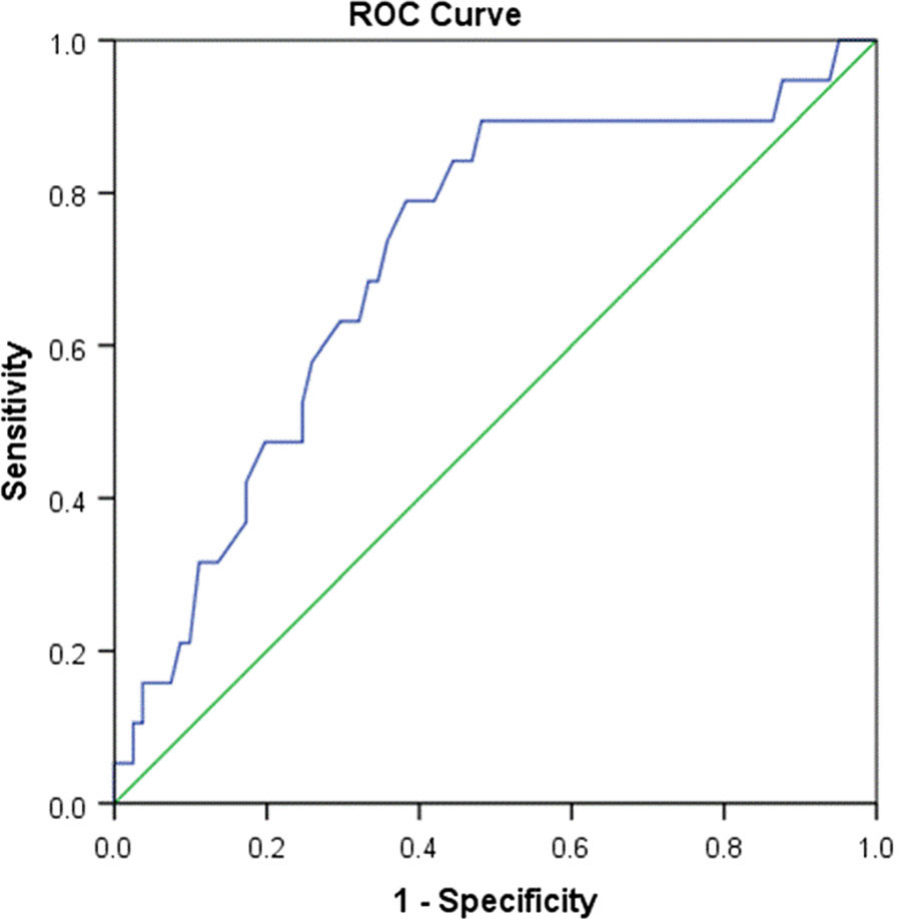
ROC curve analysis of the apelin values on the seventh day for the prediction of MACE. AUC = 0.71 (95% CI, 0.58–0.84), *p* = 0.004

A receiver operating characteristic (ROC) curve plots the true positive rate against the false positive rate at different cut-off points. Table 2 presents the area under the curve values for biomarkers, and Fig. 4 presents the ROC curve for apelin-12 and MACE.

**Table 2:** Area under the curve values for biomarkers

Biomarker	AUC (95% CI)	*P*-value
Apelin 12 on the first day	0.52 (0.37–0.67)	0.71
Apelin 12 on the seventh day	0.71 (.58–0.84)	0.004
Troponin I on the first day	0.48 (0.33–0.63)	0.8
Troponin I on the seventh day	0.41 (0.27–0.55)	0.25
Creatine kinase	0.48 (0.33–0.63)	0.84
Creatine kinase-MB	0.58 (0.43–0.73)	0.27
NT-proBNP	0.49 (0.33–0.64)	0.88
C-reactive protein	0.54 (0.39–0.69)	0.56

Abbreviations: *NT-proBNP* N-terminal pro b-type natriuretic peptide

## Discussion

In this study, we investigated the influence of apelin-12 on troponin levels and the rate of MACE in STEMI patients. The present study showed association between apelin-12 and troponin in the acute phase of myocardial infarction, whereas rates of MACE after STEMI were expected based on values of apelin-12 in the non-acute phase. The findings of other studies have demonstrated efficiency of myocardial protection induced by apelin limiting myocardial infarction and its action as a regulatory peptide increasing cadiac contractility [30, 31].

In the acute phase of myocardial infarction, the atheromatous plaque in the lumen suffers from complete or incomplete acute block-up, which results in ischaemia in the myocardium. During hypoxia (24 h, 2% O2), apelin gene expression and secretion are increased through the activation of hypoxia inducible factor (HIF) [10, 32, 33]. Hypoxia requires a functional mitochondrial electron transport chain for the inhibition of prolyl hydroxylases and HIF stabilization [34]. Under anoxic conditions, the stabilization of HIF is preserved due to the lack of a functioning mitochondrial respiratory chain, and thus, the apelin gene is neither expressed nor is its expression increased [35].

Apelin activates its sites on troponin I, regulating Ca^2+^ sensitivity and ATPase activity in the myocardium through protein kinase C [23, 24]. During ischaemia/infarction, phosphorylated TnI levels, Ca^2+^ sensitivity and ATPase activity are decreased in the myocardium, and subsequently, the elevated Ca^2+^ levels activate protease I (caplain I), leading to the proteolytic degradation of troponins and ventricular dysfunction [25–28]. The degree of association between apelin-12 and troponin I in the first day of the acute phase of STEMI was analysed with Spearman’s correlation = −0.40 (*p* < 0.001). The relationship between apelin-12 and troponin I was analysed by a regression analysis, predicting one variable from the other (Fig. 2).

The loss of apelin (APLN) clearly compromises the activation of the protective Akt/PI3K and extracellular signal-regulated kinase 1/2 (Erk1/2) signalling pathways both in vivo and ex vivo, resulting in increased myocardial damage and worsened heart function [36]. Low values of apelin in the non-acute phase of STEMI were associated with high rates of MACE after STEMI, with variability in the apelin values on the seventh day (Kruskal-Wallis test) based on major adverse cardiac events (MACE) that was significantly different (*p* < 0.012). A cut-off value for apelin-12 levels on the seventh day was set for the survival analysis, and a Kaplan-Meier curve was generated; the log-rank test for differences in survival resulted in a *p* value of 0.002. ROC curve analysis showed that the area under the curve was significantly different, with a *p* value of 0.004 (Fig. 4 and Table 2).

The vision for the future is potential utility of apelin-12 measurement in STEMI patients as an additional risk stratification tool and possibility for the usage as a therapy.

We were aware of some limitations to our study. This observational study had a relatively limited number of patients. Apelin levels and correlation with troponin in non-STEMI acute chest pain differential admissions to the emergency department would be interesting as control group.

## Conclusion

Apelin-12 influences troponin I levels in the acute phase of STEMI, whereas during the non-acute phase, low apelin levels were associated with a high rate of MACE.

### Take home message

Table 3 presents take home messages.

**Table 3:** Take home messages

Apelin-12 influences troponin I levels in the acute phase of STEMI
A high rate of MACE after STEMI was characterized with low values of apelin during non-acute phase
n the future, potential utility of apelin-12 measurement in STEMI patients as an additional risk stratification tool and possibility for the usage as a therapy

## Abbreviations

Akt/PI3K: phosphatidylinositol 3-kinase/protein kinase B
APJ: Angiotensin receptor like-1
APLN: Apelin
ATPase: Adenosine triphosphatase
DAG: Diacylglycerol
ECG: Electrocardiography
EDTA: Ethylenediaminetetraacetic acid
ELISA: Enzyme-linked immunosorbent assay
Erk: Extracellular signal-regulated kinases
HIF: Hypoxia inducible factor
IP3: Inositol trisphosphate
LAD: Left anterior descending artery
LCx: Left circumflex
LMA: Left main artery
MACE: Major adverse cardiac events
NCX: Na^+^/Ca^2+^ exchanger
NHE: Na^+^/H^+^ exchanger
NT-proBNP: N-terminal pro b-type natriuretic peptide
PIP2: Phosphatidyl inositol bisphosphate
PKC: Protein kinase C
PLB: Phospholamban
PLC: Phospholipase C
RCA: Right coronary artery
ROC: Receiver operating characteristic
RYRs: Ryanodine receptors
SD: Standard deviation
SERCA: Sarco/endoplasmic reticulum Ca^2+^ ATPase
STEMI: ST-Segment elevation myocardial infarction
